# Acquired resistance to everolimus in aromatase inhibitor-resistant breast cancer

**DOI:** 10.18632/oncotarget.25133

**Published:** 2018-04-20

**Authors:** Mariko Kimura, Toru Hanamura, Kouki Tsuboi, Yosuke Kaneko, Yuri Yamaguchi, Toshifumi Niwa, Kazutaka Narui, Itaru Endo, Shin-Ichi Hayashi

**Affiliations:** ^1^ Department of Molecular and Functional Dynamics, Tohoku University Graduate School of Medicine, Sendai, Japan; ^2^ Department of Gastroenterological Surgery and Clinical Oncology, Yokohama City University Graduate School of Medicine, Yokohama, Japan; ^3^ Division of Breast and Endocrine Surgery, Department of Surgery, Shinshu University School of Medicine, Nagano, Japan; ^4^ Research Institute for Clinical Oncology, Saitama Cancer Center, Ina-machi, Japan; ^5^ Department of Breast and Thyroid Surgery, Yokohama City University Medical Center, Yokohama, Japan

**Keywords:** breast cancer, estrogen receptor, endocrine resistance, mTOR inhibitor, everolimus

## Abstract

We previously reported the establishment of several types of long-term estrogen-depleted-resistant (EDR) cell lines from MCF-7 breast cancer cells. Type 1 EDR cells exhibited the best-studied mechanism of aromatase inhibitor (AI) resistance, in which estrogen receptor (ER) expression remained positive and PI3K signaling was upregulated. Type 2 EDR cells showed reduced ER activity and upregulated JNK-related signaling. The mTOR inhibitor everolimus reduced growth in cells similar to Type 1 EDR cells. The present study generated everolimus-resistant (EvR) cells from Types 1 and 2 EDR cells following long-term exposure to everolimus *in vitro*. These EvR cells modeled resistance to AI and everolimus combination therapies following first-line AI treatment failure. In Type 1 EvR cells, everolimus resistance was dependent on MAPK signaling; single agents were not effective, but hormonal therapy combined with a kinase inhibitor effectively reduced cell growth. In Type 2 EvR cells, ER expression remained negative and a JNK inhibitor was ineffective, but a Src inhibitor reduced cell growth. The mechanism of acquired everolimus resistance appears to vary depending on the mechanism of AI resistance. Strategies targeting resistant tumors should be tailored based on the resistance mechanisms, as these mechanisms impact therapeutic efficacy.

## INTRODUCTION

Endocrine therapy plays an important role in estrogen receptor (ER)-positive breast cancer treatment [1], but resistance to these drugs has become a major clinical problem [2, 3]. Aromatase inhibitors (AIs) are currently the most widely used agents in the treatment of ER-positive postmenopausal breast cancer [4], and AI resistance is an important issue [5, 6]. “Crosstalk” between ER and growth factor receptor pathways is associated with acquired resistance to endocrine therapy [7]. Several groups have explored mechanisms of AI resistance in estrogen-depleted-resistant (EDR) ERα-expressing breast cancer cells [8–11] using whole cells cultured long-term in estrogen-depleted media. These reports suggested that resistant cells acquired estrogen hypersensitivity through crosstalk with the MAP-kinase or PI3K/Akt/mTOR pathways and the involvement of membrane-associated ERs [10–12]. However, mechanistic details are still lacking, and additional mechanisms may also be associated with resistance [13]. Our previous study analyzed refractory specimens using an adenovirus estrogen response element-green fluorescent protein (ERE-GFP) assay [14]. We found that ER activity and sensitivity to anti-estrogens varied among patients. We established several MCF-7 cell sub-lines by isolating single colonies under various conditions mimicking AI treatment. MCF-7 cells were stably transfected with an ERE-GFP on ER activation [15], and ER activity in living cells was assessed via fluorescence. Using these cells (named MCF-7-E10 cells), we established several clones mimicking AI resistance [16]. The present study employed two of our cell lines [17]: type 1 EDR cells show upregulated PI3K/Akt/mTOR signaling and constitutive ER overexpression without estrogen, and type 2 EDR cells exhibit low ER expression (ERE-GFP negative) and upregulated receptor tyrosine kinases (RTKs)/JNK signaling (Supplementary Figure 1).

Clinically, many breast cancers with acquired resistance to AIs retain ER expression. Thus, novel therapeutic strategies target both ER and other signaling pathways in endocrine-resistant breast cancer cells. PIK3CA mutations occur in 28–47% of ER-positive breast cancers [17, 18] and PI3K/Akt/mTOR signaling is often upregulated in endocrine-resistant cells [19]. Drugs targeting this pathway have shown promising results in combination with AIs or anti-estrogens [20]. Currently, the most clinically advanced PI3K/Akt/mTOR-targeting agent for treatment of ER-positive metastatic breast cancer is the mTOR inhibitor, everolimus.

The BOLERO-II study, a large randomized phase III trial in postmenopausal nonsteroidal AI-resistant ER-positive breast cancer patients [21], showed significant and clinically relevant improvements in progression-free survival with everolimus in combination with exemestane [22]. Clinical benefits were due to better disease control [23]. However, biomarkers predicting patients who would benefit from everolimus have not been identified, despite comprehensive sequence analyses [24]. Additionally, treatments for tumors refractory to everolimus have not been established.

In the present study, everolimus-resistant (EvR) cells were generated from our previously established Type 1 and 2 EDR cells following long-term exposure to everolimus *in vitro*. These EvR cells modeled resistance to AI and everolimus combination therapy following first-line AI treatment failure. Using these cells, we investigated mechanisms of resistance to everolimus.

## RESULTS

### Sensitivity to everolimus *in vitro* and *in vivo*

**Figure 1:**
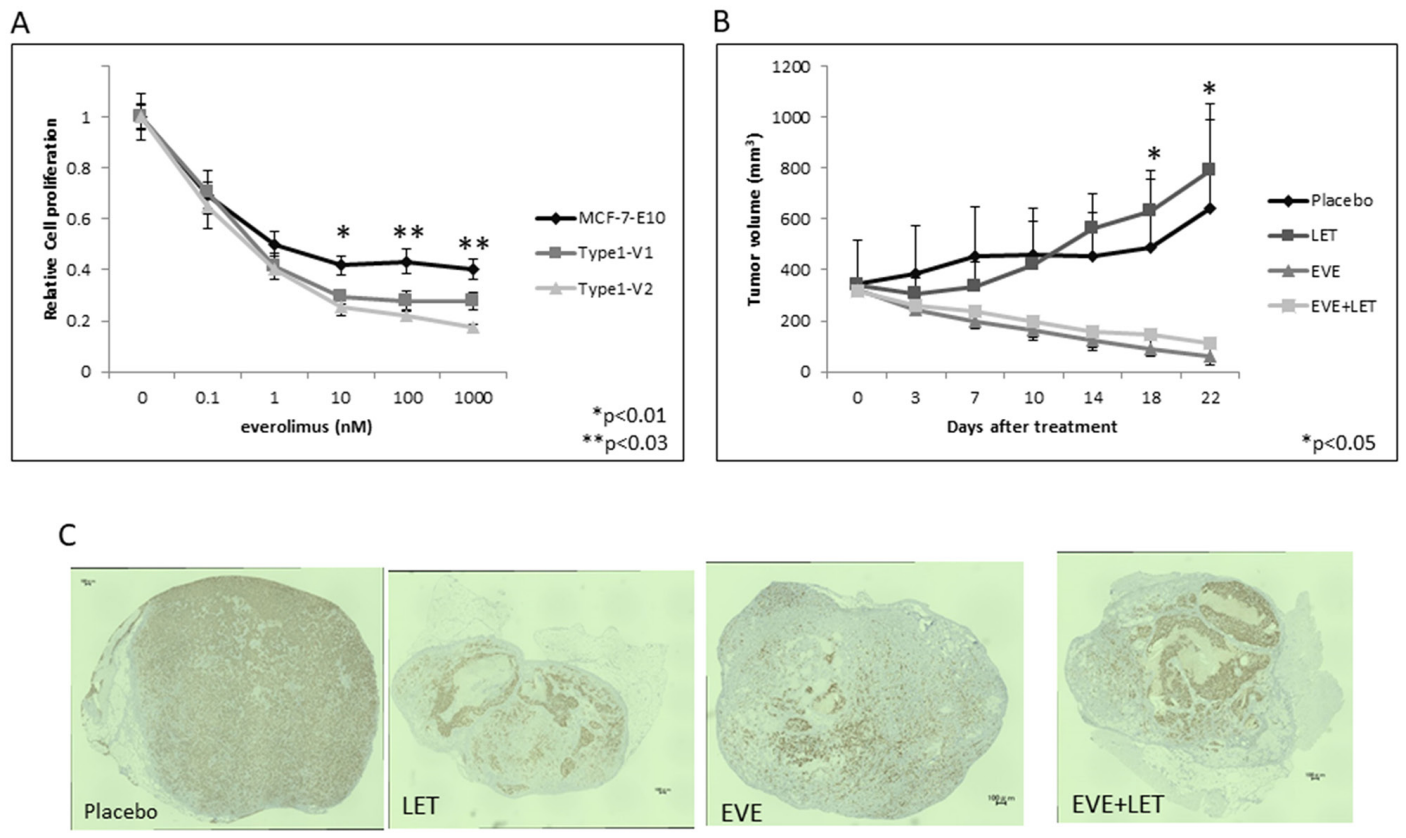
Effects of everolimus in various EDR cell types *in vitro* and *in vivo* Effect of everolimus on MCF-7-E10 cells and two Type 1 EDR cell variants (V1, V2). **(A)** Everolimus dose-dependently suppressed cell proliferation. Data are shown as means ± SD of three independent experiments. ^*^P<0.01, ^**^P<0.03 between parental MCF-7-E10 and Type 1 V1, V2 cells. Treatment effects on tumor growth *in vivo*. **(B)** OVX scid mice were inoculated with Type 1 EDR cells. When tumors reached >300 mm^3^, mice were treated daily with placebo, letrozole (LET), everolimus, or everolimus with LET for 21 d. Tumors were measured twice weekly, and tumor size was averaged for each treatment group. ^*^P<0.05 between treatment groups and placebo or LET-treated mice. Immunohistochemical staining for ER expression in enucleated tumors from the four treatment groups. **(C)** Almost all cells of placebo-treated tumors showed strong ER expression. Everolimus and/or LET-treated tumors showed lower ER expression, with necrosis in everolimus-treated tumors.

Everolimus dose-dependently inhibited cell growth *in vitro* in parental MCF-7-E10 cells and in our Type 1 EDR cell variants 1 (Type 1-V1) and 2 (Type 1-V2), which have EDR properties similar to other highly reported EDR cell lines. Everolimus was more effective in the EDR cells than in the parental MCF-7-E10 cells, but this difference was not significant (Figure 1A). We used the Type 1-V1 EDR cell line to study xenograft tumor sensitivity to placebo, letrozole, everolimus, or a combination of everolimus and letrozole. Placebo- and letrozole-treated groups showed no tumor reductions during the 21-d treatment period. In contrast, everolimus-treated tumors were reduced in size, but there was no difference in tumor response between treatments with and without letrozole (Figure 1B). ER expression was reduced in tumors in all treatment groups except the placebo. Immunohistochemical (IHC) analyses showed ERα positivity at 88.6%, 89.0%, 51.2%, and 56.8% in the placebo, letrozole, everolimus, and combination treatment groups, respectively (Figure 1C).

### Establishment of everolimus-resistant EDR cell lines

**Figure 2:**
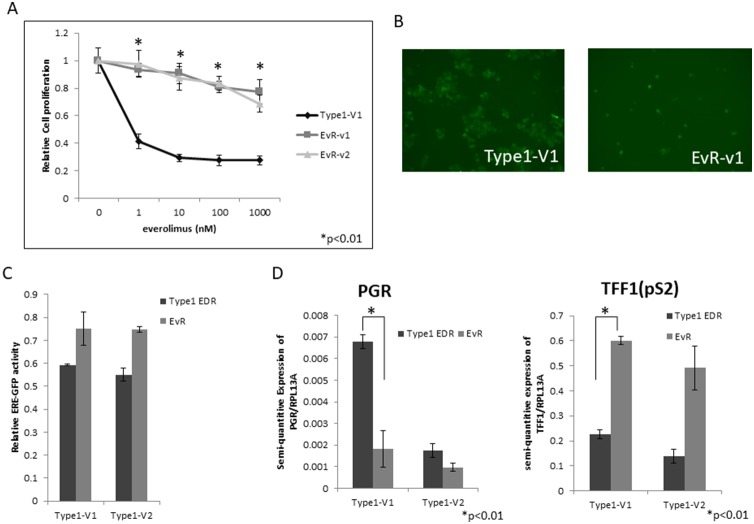
EvR cell establishment and characterization Effect of everolimus on Type 1 EDR cells (using V1) and everolimus-resistant variants established from Type 1 EDR-V1 (v1, v2) cells. **(A)** Everolimus-resistant cell (EvR) variants were cloned as follows: after long-term everolimus exposure and culture in a single dish, surviving cells were isolated and harvested. Highly proliferated variants were selected (variants 1 and 2). Everolimus suppressed cell proliferation in parental EDR cells, but not EvR cells. All data are shown as means ± SD of three independent experiments. ^*^P<0.01 between parental Type 1 EDR and Type 1 EvR v1, v2 cells. GFP positivity was maintained after resistance to everolimus was acquired in Type 1 EDR cells (EvR-v1). **(B)** Comparison of luciferase assay in EDR and EvR cells. **(C)** Both Type 1 EDR (V1, V2) and EvR (generated individually from Type 1 EDR-V1 and V2) cells showed high estrogen response element-green fluorescent protein activity. *PGR* and *TFF1* (*pS2*) expression in Type 1 EDR (V1, V2) and EvR cells. **(D)** Data are shown as means ± SD of three independent experiments. ^*^P<0.01 between parental Type 1 EDR and Type 1 EvR v1 cells.

Repeated treatments of harvested EDR cells with 1 μM everolimus nearly eliminated all Type 1 cells (Supplementary Figure 2). Therefore, to generate everolimus-resistant cells, we gradually increased everolimus concentrations in EDR cell cultures, isolated single cells that survived after several months, and harvested those clones as sub-cell lines. Several of these Type 1 EDR cell variants gained resistance to everolimus (became EvR cells) (Figure 2A, Supplementary Figure 3). Our previous report [14] showed that Type 1 EDR cells were GFP positive for ER expression, and the generated EvR cells were also GFP positive (Figure 2B). There was no difference in GFP intensity between the parental Type 1 EDR and EvR cells in variant 1, but in variant 2 Type 1, EvR cell GFP intensity was less than that of the parental cells. Additionally, ER expression as shown by ERE luciferase assays was the same as that of GFP (Figure 2C). Expression of estrogen receptor 1 (*ESR1*), which encodes ERα, was higher in EvR cells than in the parental EDR Type 1 cells (Supplementary Figure 4). Progesterone receptor (PGR) and trefoil factor 1 (TFF1 or pS2), are both involved in downstream ERα signaling. *PGR* was downregulated in EvR cells compared to parental EDR cells, but *TFF1* (*pS2*) was upregulated in EvR cells (Figure 2D).

**Figure 3:**
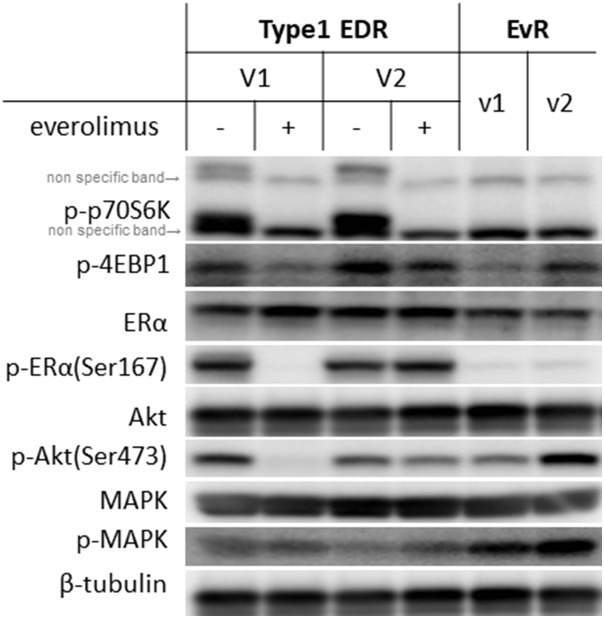
Protein levels in Type 1 EDR-V1 and V2 cells and in EvR (v1, v2) cells generated from EDR-V1 cells Downstream of mTOR, p-p70S6K was not observed with everolimus treatment in any cell type. p-4EBP1 levels varied. ERα was expressed in each cell type, but p-ER (Ser167) was not observed in EvR cells. Akt was expressed in each cell type, but p-Akt (Ser473) expression varied. MAPK was expressed in each cell type, with higher p-MAPK levels in EvR cells than in parental EDR cells.

We examined levels of various proteins in the mTOR and ER cascade (Figure 3). While total protein levels were generally the same between EvR and parental EDR cells, everolimus resistance altered phosphorylation patterns. Downstream of mTOR, p70S6K, and 4EBP1 remained suppressed with everolimus in both the parental and EvR cells. p-MAPK expression was greater in EvR cells than in EDR cells.

### Agents effective against everolimus-resistant cells

**Figure 4:**
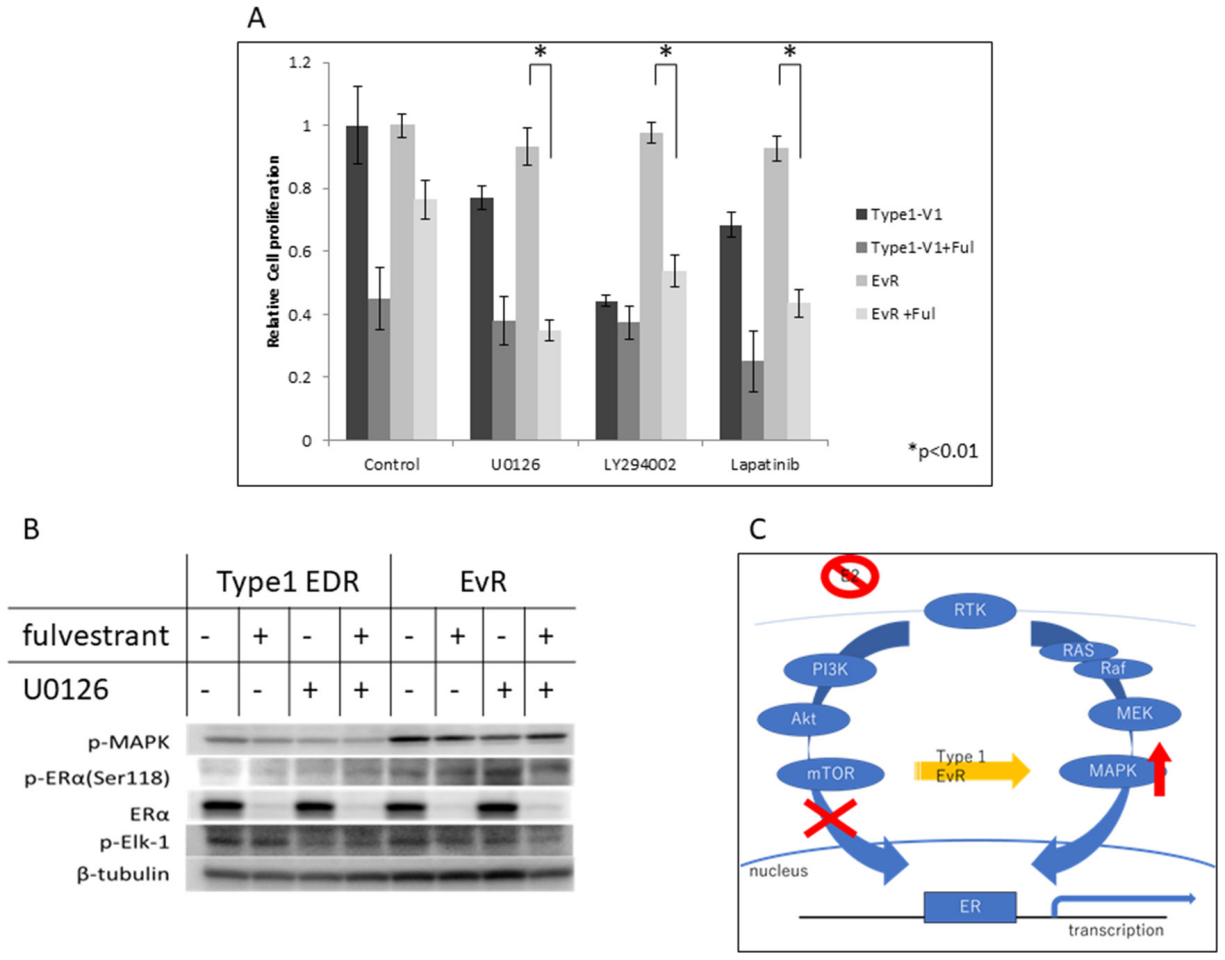
Effects of various agents in Type 1 EDR-V1 and EvR-v1 (from EDR-V1) cells Agent concentrations were as follows: fulvestrant, 100 nM; MEK inhibitor U0126, 1 μM; PI3K inhibitor LY294002, 1 μM; lapatinib, 1 μM. **(A)** Data are shown as means ± SD of three independent experiments, relative to cells treated with vehicle. ^*^P<0.01 in kinase inhibitor monotherapy and in combination with fulvestrant in EvR. Protein levels in Type 1 EDR-V1 and EvR-v1 cells treated with fulvestrant (100 nM) or U0126 (1 μM). **(B)** Protein was extracted 24 h after each agent was added. p-MAPK was not affected by either treatment, but fulvestrant inhibited total ERα expression in each cell type. p-Elk-1, downstream of ER, was unaffected by treatment. Mechanism of acquired resistance to everolimus in Type 1 cells. **(C)** After resistance to everolimus was acquired, cell proliferation depended on the MEK/MAPK cascade, and not the PI3K/Akt/mTOR cascade.

We attempted to identify agents that effectively inhibited EvR cell growth. In contrast to parental EDR cells, anti-ER agents, such as fulvestrant and tamoxifen, were not effective in EvR cells (Figure 4A; tamoxifen data not shown, but same as fulvestrant). Various kinase inhibitors used alone or in combination with fulvestrant are shown in Figure 4A. The MEK inhibitor U0126, PI3K inhibitor LY294002, and lapatinib were not effective in EvR cells when administered as single agents. However, these agents in combination with fulvestrant suppressed cell growth similarly in EvR and parental EDR cells. U0126 with fulvestrant was the most effective combination treatment in EvR cells (Figure 4A). Fulvestrant suppressed total ERα in both EDR and EvR cells, although p-ER (Ser118)-related MEK signaling was upregulated in EvR cells. There was no difference in p-MAPK expression with or without the MEK inhibitor (Figure 4B). Figure 4C shows the mechanism of acquired resistance to everolimus in Type 1 cells.

### ER-negative EvR cells from Type 2 EDR cells

**Figure 5:**
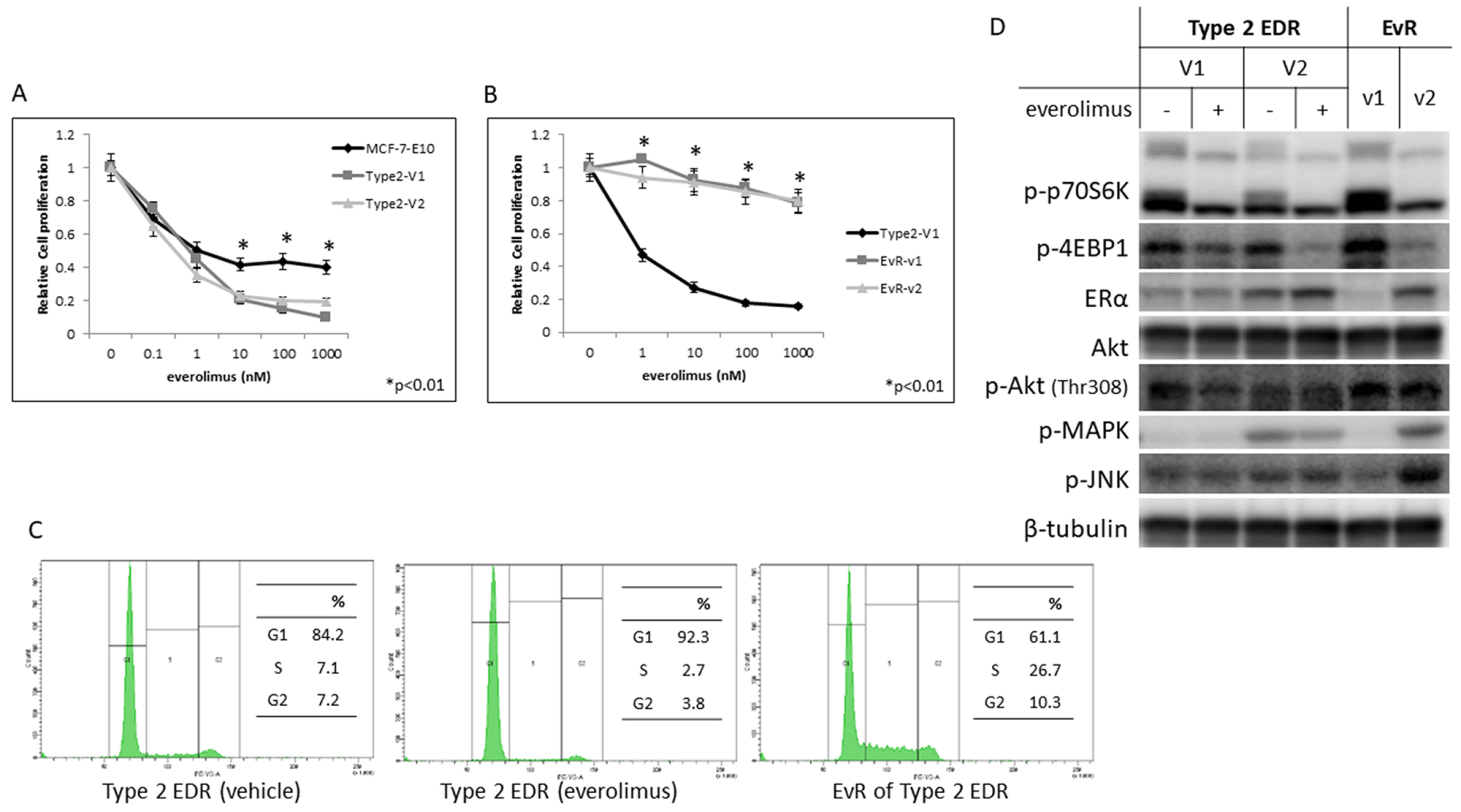
Effect of everolimus on MCF-7-E10 cells and two variants (V1, V2) of Type 2 EDR cells Everolimus dose-dependently suppressed cell proliferation. **(A)** ^*^P<0.01 between parental MCF-7-E10 and Type 2 V1 and V2 cells. Effects of everolimus on Type 2 EDR (using V1) cells and everolimus-resistant variants established from Type 2 EDR-V1 (EvR-v1, v2) cells. **(B)** EvR variants were isolated and cloned using the same methods as Type 1 cells. Everolimus suppressed cell proliferation in parental EDR cells, but not EvR cells. ^*^P<0.01 between parental Type 2 EDR and Type 2 EvR v1, v2 cells. Cell cycle fluorescence-activated cell sorter analysis. **(C)** Type 2 EDR (V1) cells treated with vehicle (left) or everolimus (20 nM) for 24 h (middle). EvR-v1 cells under the usual harvested conditions (right). EvR cells were found to be in S phase more frequently than parental EDR Type 2 cells. Protein levels in Type 2 EDR (V1 and V2) cells and of EvR-v1 and v2 from Type 2 EDR (V1) cells. **(D)** Protein was extracted 24 h after everolimus (20 nM) was added to Type 2 EDR cells. EvR cell proteins were extracted under sub-confluent conditions. Everolimus suppressed p-p70S6K and p-4EBP1 levels in parental EDR cells, but increased phosphorylation of these proteins in EvR variants. In Type 2 EDR cells, ER expression differed among the variants; variants with higher ER expression were similar to Type 1 EvR cells. p-JNK expression also varied among the variants. Data are shown as means ± SD of three independent experiments.

We previously reported the generation of two EDR cell types: cells that either maintained (Type 1) or lost (Type 2) ER expression. Type 2 EDR cells were more sensitive to everolimus than were the parental cells (Figure 5A) and gained everolimus resistance earlier than did Type 1 EDR cells (Figure 5B, Supplementary Figure 2). In the cell cycle assay, everolimus decreased the number of parental EDR cells in S phase, but increased the number of EvR cells in S phase (Figure 5C, Supplementary Figure 5). Protein phosphorylation patterns also differed between the two variants (Figure 5D).

**Figure 6:**
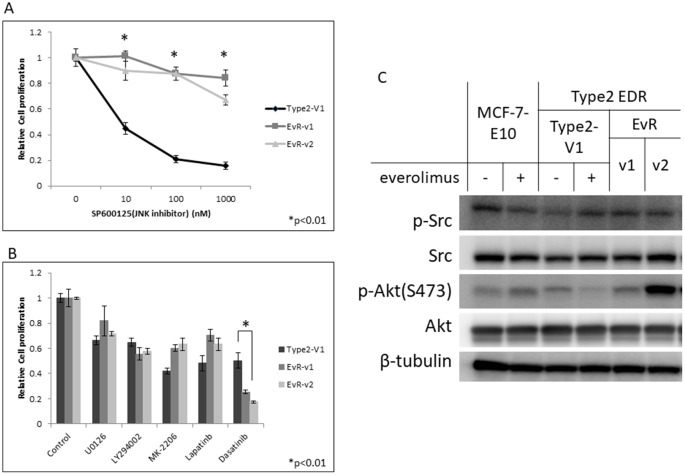
Effect of the JNK inhibitor, SP600125, on Type 2 EDR (using V1) cells and everolimus-resistant variants generated from Type 2 EDR-V1 cells (EvR-v1, v2) **(A)** SP600125 suppressed cell proliferation in the parental EDR cells, but not in EvR cells. ^*^P<0.01 between parental Type 2 EDR and Type 2 EvR v1, v2 cells. Responses of various agents in Type 2 EDR-V1 and EvR-v1 and v2 (from Type2 EDR-V1) cells. **(B)** Agent concentrations were as follows: MEK inhibitor U0126, 1 μM; PI3K inhibitor LY294002, 1 μM; Akt inhibitor MK-2206, 1 μM; lapatinib, 1 μM; Src inhibitor dasatinib, 1 μM. Except for dasatinib, cell responses to agents were the same. ^*^P<0.01 between parental Type 2 EDR and Type 2 EvR v1, v2. Protein levels in treated MCF-7-E10, Type 2 EDR (V1), and EvR-v1 and v2 of Type 2 EDR cells. **(C)** Protein was extracted 24 h after everolimus (20 nM) was added to parental EDR cells. EvR cell proteins were extracted under sub-confluent conditions. Dasatinib reduced cell growth, but total Src and p-Src levels were the same between cell types. Everolimus did not affect Akt and p-Akt (Ser473) levels. Data are shown as means ± SD of three independent experiments.

The JNK inhibitor effectively reduced cell growth in parental Type 2 EDR cells, in which the JNK signaling pathway is upregulated, but did not inhibit EvR cell growth (Figure 6A). Of the kinase inhibitors tested, only the Src inhibitor, dasatinib, inhibited Type 2 EvR cell growth (Figure 6B). Total and phosphorylated Src protein levels were nearly the same between parental EDR and EvR cells (Figure 6C).

## DISCUSSION

The BOLERO-II trial showed that everolimus is an effective treatment for ER-positive postmenopausal metastatic breast cancer when used in combination with the steroidal AI, exemestane [21]. Everolimus has thus become increasingly important in breast cancer treatment strategies [25]. Several hormonal therapies are currently available for breast cancer patients, but the most effective treatment regimen order is not clear. We previously elucidated several EDR mechanisms, and different treatments were needed for each resistant cell line according to their biological characteristics [16]. Our present study confirmed that everolimus effectively inhibited cell growth in two distinct EDR cell lines. Type 1 EDR cells are a major cell population with EDR characteristics mimicking AI resistance, and several studies have reported that everolimus was effective in this cell type [26–28]. Everolimus was also effective in ER-negative Type 2 EDR cells, in which hormonal therapy was no longer effective. Thus, even if ER-positive breast cancers acquire resistance, our findings imply that everolimus would likely still be effective to some extent.

Through long-term everolimus exposure, we established everolimus-resistant cell lines with and without estrogen depletion resistance. This suggests that there might be different everolimus resistance mechanisms. In Type 1 EDR-EvR cells, acquired resistance to everolimus does not affect ER expression. Despite this, *PGR* and *TFF1* (*pS2*) downstream of *ESR1* were downregulated. These results imply that everolimus resistance deregulates ER signaling. p-p70S6K was suppressed in both everolimus-resistant cells and in parental cells treated with everolimus.

Currently, no treatment approach has been established to follow everolimus plus exemestane failure, and the appropriate order of hormonal therapy regimens prior to chemotherapy has not been determined. Combined blockade of ER, various growth factor receptors, and intracellular signaling pathways appears to be important for achieving crosstalk between pathways, and such combination therapies have been studied preclinically and clinically [29, 30]. Resistance to hormonal agents and kinase inhibitors can likely occur through multiple mechanisms, and suitable treatments should be matched to individual resistance mechanisms. Our study clearly showed that ER-positive EDR cells (Type 1) no longer responded to single hormonal therapy agents, but these agents were useful to varying degrees when combined with kinase inhibitors. Therefore, if ER positivity is retained and ER signaling remains partly effective, inhibition of this pathway would still be meaningful in those cell types.

Clinical trials based on this hypothesis have employed sequential regimens in ER-positive metastatic breast cancer [30]. Our EDR cells that lost ER expression (Type 2) gained everolimus resistance more quickly than did Type 1 cells, and Type 2 EvR cells remained ER negative. This suggests that ER expression and signaling might delay resistance to everolimus. Although the JNK inhibitor effectively inhibited EDR Type 2 cell growth, Type 2 EvR cells were unresponsive. The pan-Src inhibitor, dasatinib, was more effective in these cells than the JNK inhibitor. We were unable to elucidate the molecular mechanism here, as Src and p-Src levels did not differ between the two cell types. However, the ER-Src axis appears important in metastatic ER-positive breast cancer [31, 32]. ER-negative converted breast cancer differs molecularly from triple-negative breast cancer, and dasatinib might be more useful in populations with endocrine-resistant breast cancer.

We also assessed several chemotherapy agents in the EDR and EvR cells, but there were no differences in responses to these treatments (Supplementary Figure 6), indicating that acquired resistant to everolimus or AIs was not a concern in chemotherapy. Microarray analyses using these cells showed that cell cycle acceleration-related factors were upregulated in EvR cells more than in EDR cells (data not shown). In agreement with these findings, our flow cytometry results suggested that G1 arrest, an effect of everolimus, might not occur in EvR cells [33].

Various mechanisms of resistance to hormonal or kinase inhibitor agents likely lead to different clinical outcomes. More clarity is needed regarding the underlying mechanisms affecting cell growth and survival following each anti-breast cancer treatment regimen. Elucidation of these intracellular molecular mechanisms could contribute to development of more effective treatments against ER-positive metastatic breast cancer.

## MATERIALS AND METHODS

### Reagents

Everolimus (RAD001) was kindly provided by Novartis Pharma KK (Basel, Switzerland). U0126 was purchased by Cell Signaling Technology Inc. (Danvers, MA, USA). Western blotting antibodies included: ERα (H-184) from Santa Cruz Inc. (Santa Cruz, CA, USA); p-p70S6K (#9204), p70S6K (#9202), p-4EBP1 (#2855), 4EBP1 (#9452), p-Akt (Ser473) (#4060), p-Akt (Thr308) (#2965), Akt (#4691), p-ER (Ser167) (#2514), p-ER (Ser118) (#2515), p-p44/42 MAPK (Thr180/Tyr182) (#4370), p44/42 MAPK (Erk1/2) (#4695), p-Elk-1 (Ser383) (#9181), p-JNK (#4668), p-Src (#2101), Src (#2191) and β-tubulin (#2145) from Cell Signaling Technology Inc.

### Cell lines and culture

MCF-7-E10 cells were stably transfected with an ERE-GFP reporter plasmid as previously described [14, 15]. Type 1 and 2 EDR cells were established as cloned variants from MCF-7-E10 cells under conditions of long-term estrogen depletion [17]. Type 1 EDR cells showed ER overexpression and PI3K/Akt/mTOR pathway upregulation. Type 2 EDR cells showed reduced ER expression and upregulated JNK-related signaling. MCF-7-E10 cells were cultured in RPMI 1640 (Sigma-Aldrich) supplemented with 5% fetal calf serum (FCS; Tissue Culture Biologicals, Turale, CA, USA) and 1% penicillin-streptomycin (Sigma-Aldrich). Type 1 and 2 EDR cells were maintained in phenol red-free RPMI supplemented with 5% dextran-coated charcoal treated FCS (DCC-FCS; an estrogen and other steroid hormones-depleted serum), and 1% penicillin-streptomycin. Everolimus resistant (EvR) cells were individually established from Type 1 and 2 EDR cells under the same culture conditions as each parental EDR cell line, but with constitutive exposure to everolimus. All cells were incubated at 37°C in a humidified atmosphere with 5% CO_2_. The characteristics of these cells did not change with passage number.

### *In vitro* proliferation assay

Parental MCF-7-E10 cells were prepared after three days in steroid-depleted medium. Cells were seeded in triplicate at 20,000 cells/well into 24-well plates. At the same time, everolimus at 5 step concentrations (1μM, 100 nM, 10 nM 1 nM, 100 pM) was added to the wells to obtain a dose-response curve.. Control wells without everolimus were also seeded. Four days later, cells were harvested using trypsin and counted using a Sysmex CDA-500 automated cell counter (Sysmex, Kobe, Japan).

### Luciferase reporter assay

The estrogen response element reporter plasmid, ERE-tk-Luci, was used as described previously [14, 15]. The control vector, pRL-TK (Promega, Madison, WI, USA), was used as an internal control for transfection efficiency. The luciferase assay was performed according to a previous report [17]. Cells were cultured in a steroid-depleted medium for three d before transfection using the TransIT reagent (Mirus, Madison, WI, USA), and luciferase activity was measured using the Dual-Luciferase Reporter Assay System (Promega).

### Western blot analysis

Proteins were extracted using Complete Lysis-M (Roche, Indianapolis, IN, USA). Extracts were subjected to SDS-PAGE (Super Sep Ace 7.5, 10 or 15%, Wako Pure Chemical Industries, Osaka, Japan) and transferred onto a membrane (Amersham Hybond-P PVDF Membrane, GE Healthcare, Buckinghamshire, UK). Primary and secondary antibodies are listed above in the Reagents subsection. Antibody-protein complexes were detected using Immun-Star™ AP substrate (Bio-Rad Laboratories), and protein bands were visualized using an ImageQuant™ LAS 4000 image analyzer (GE Healthcare Bio-Sciences AB, Uppsala, Sweden).

### Flow cytometry

EDR cells were seeded into 6-cm plates. Cells were treated with vehicle, 100 nmol/L letrozole, and 0.2 or 2 nmol/L RAD001 (alone or in combination) for 24 h. Floating cells were collected and adherent cells were harvested via trypsinization. Cells were washed once with PBS and then resuspended in propidium iodide buffer. After 30 min incubation in the dark on ice, cell cycle distribution was analyzed using a flow cytometer (LSRFortessa, BD Biosciences).

### Real-time polymerase chain reaction (PCR)

Total RNA was extracted from whole cells using Isogen (Nippon Gene Co., Ltd., Toyama, Japan) according to the manufacturer's instructions. Extracted RNA (1 μg) was converted to first-strand cDNA primed with a random hexamer using an RNA PCR kit (Takara Bio Inc., Otsu, Japan), and a 2 μl aliquot was used as a template for real-time PCR. All RNA quantification was performed according to the standard protocol on an Applied Biosystems Step One real-time PCR system (Applied Biosystems Inc., Foster City, CA, USA). Target gene expression was normalized to glyceraldehyde-3-phosphate dehydrogenase (GAPDH). All PCR assays were performed at least twice, and the results shown were from samples analyzed in triplicate in one experiment. These results confirmed the reproducibility of the data obtained. Primer sequences were as follows: ESR1-forward, 5’-GAG CAG TTT GCTAAA CCA AC-3’; reverse, 5’-AGA CCG ATG TCC ATT ACA TT-3’; TFF1 (pS2)-forward, 5’-TCC CCT GGT GCT TCT ATC CTA A-3’; reverse, 5’-ACTAAT CAC CGT GCT GGG GA-3’; PGR-forward, 5’-AGC TCA CAG CGTTTC TAT CA-3’; reverse, 5’-CGG GAC TGG ATA AAT GTA TTC-3’.

### Xenografts

Experiments were performed in accordance with the United Kingdom Coordinating Committee on Cancer Research Guidelines for the welfare of animals in experimental neoplasia (2nd ed.). Six-week-old female ovariectomized (OVX) C.B-17/lcr-scid Jcl mice were obtained from CLEA Japan Inc. Animals were housed in a pathogen-free environment under controlled light and humidity conditions, and received food and water ad libitum. Intact OVX mice were inoculated with Type 1 EDR cell suspensions in Matrigel (BD Japan) at two sites in each flank (5 × 10^6^ cells/site). Tumor growth was measured using calipers twice per week. Tumor volumes were calculated as follows: (short diameter)^2^ × (long diameter)/2. Mice treated with placebo, letrozole, and/or everolimus received agents by compulsory gavage. Letrozole was dosed at 10 μg/day and everolimus at 10 mg/kg/day. Both agents and the placebo micro-emulsion were kindly provided by Novartis Pharma. Treatments began when tumors reached approximately 300 mm^3^, and each treatment group consisted of 5–6 mice. Treatment lasted for 21 d. All mice were euthanized at d 22 after measuring their tumors, and all tumors were enucleated.

### Statistical analyses

Student's *t*-tests was used to assess differences between two groups using averaged data obtained in triplicate. Data were expressed as means ± SD. P<0.03 or P<0.01 indicated statistical significance.

## SUPPLEMENTARY MATERIALS FIGURES AND TABLES


