# Supplementary material for: Acquired resistance to everolimus in aromatase inhibitor-resistant breast cancer
Source: Oncotarget. 2018 Apr 20;9(30):21468–77. doi: 10.18632/oncotarget.25133 (PMC5940386; doi:10.18632/oncotarget.25133)
Supplement: Supplementary file 1 [file oncotarget-09-21468-s001.pdf]

Acquired resistance to everolimus in aromatase inhibitor-resistant breast cancer

SUPPLEMENTARY MATERIALS

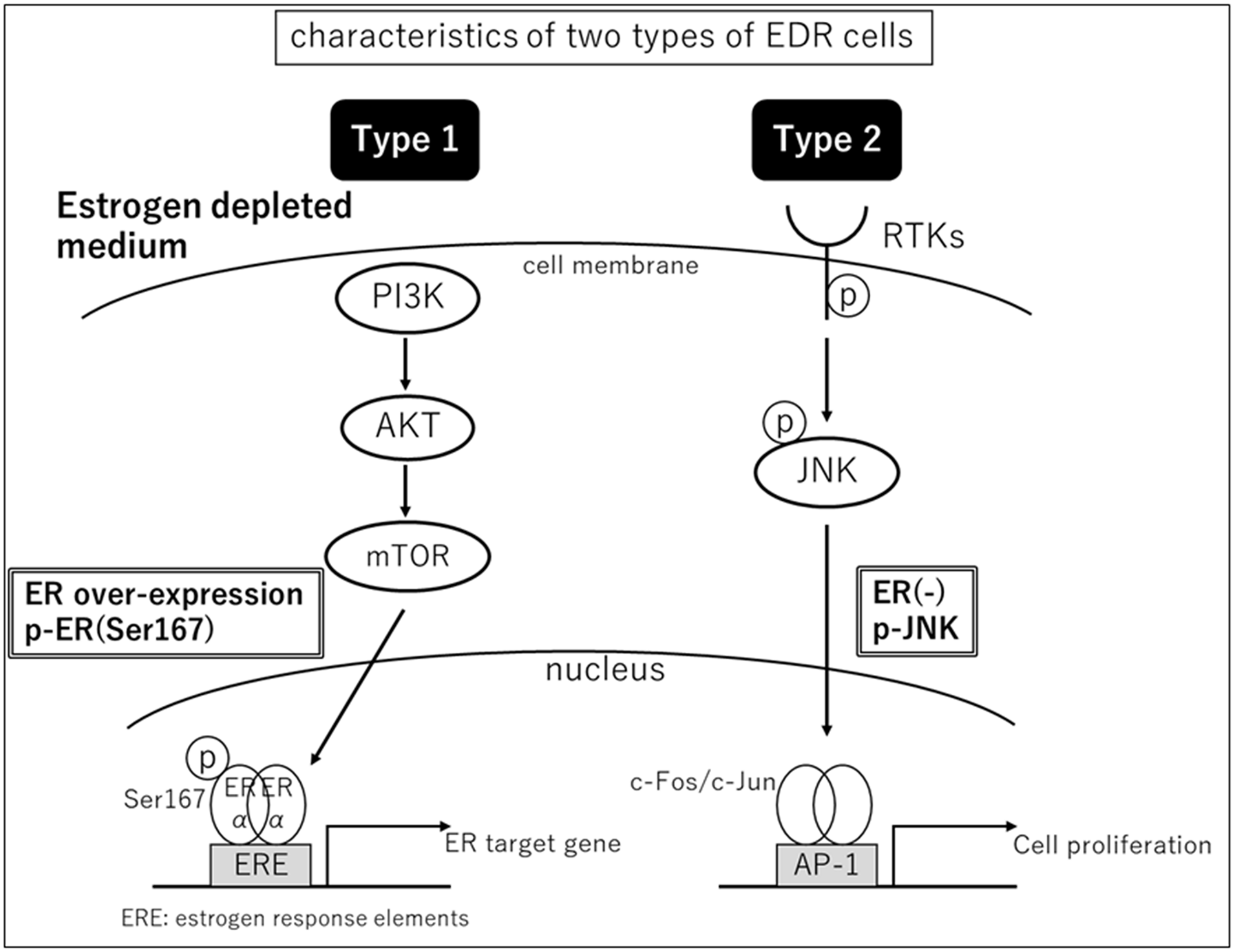

Supplementary Figure 1: Characteristics of each EDR cell.

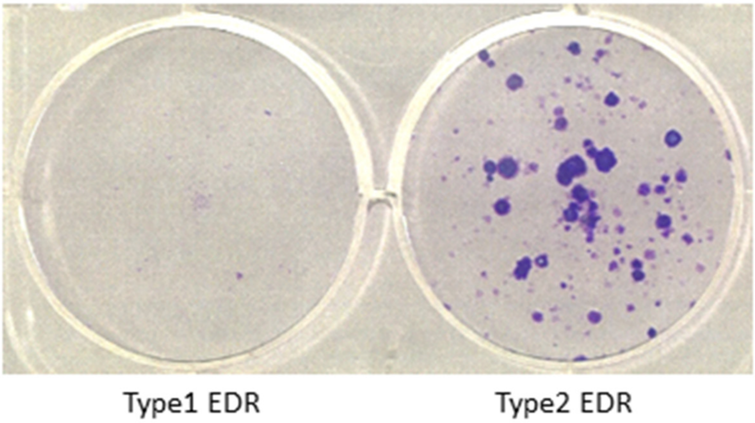

Supplementary Figure 2: Colony formation assay using Type 1 and Type 2 EDR cells in medium with everolimus (1 μM). Almost all Type 1 cells were exterminated, but Type 2 cells survived, proliferated, and produced colonies six weeks after harvest. Cell types were assessed in triplicate in six-well plates.

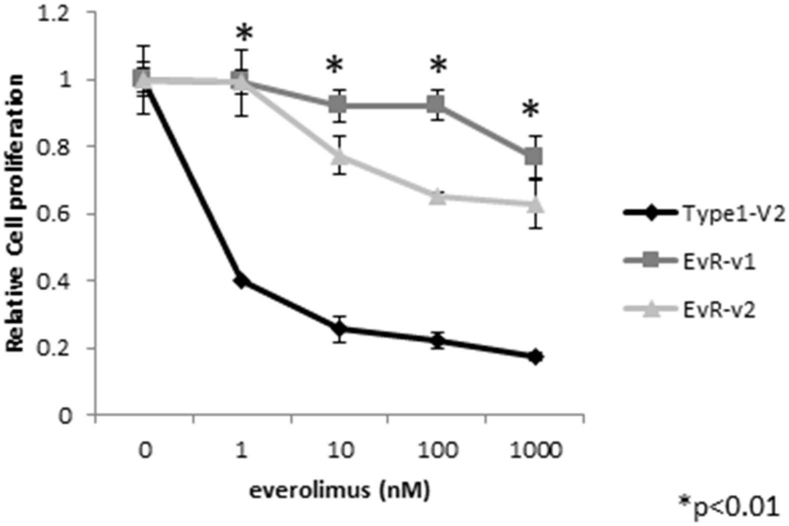

Supplementary Figure 3: Effect of everolimus on Type 1 EDR cells (using V2) and everolimus-resistant variant (EvR) cells established from Type 1 EDR-V2 (v1, v2). EvR variants were cloned in the same manner as Type 1-V1 cells. Data are shown as means ± SD of three independent experiments. *P<0.01 between parental Type 1 EDR-V2 and Type 1 Ev-V2 v1, v2 cells.

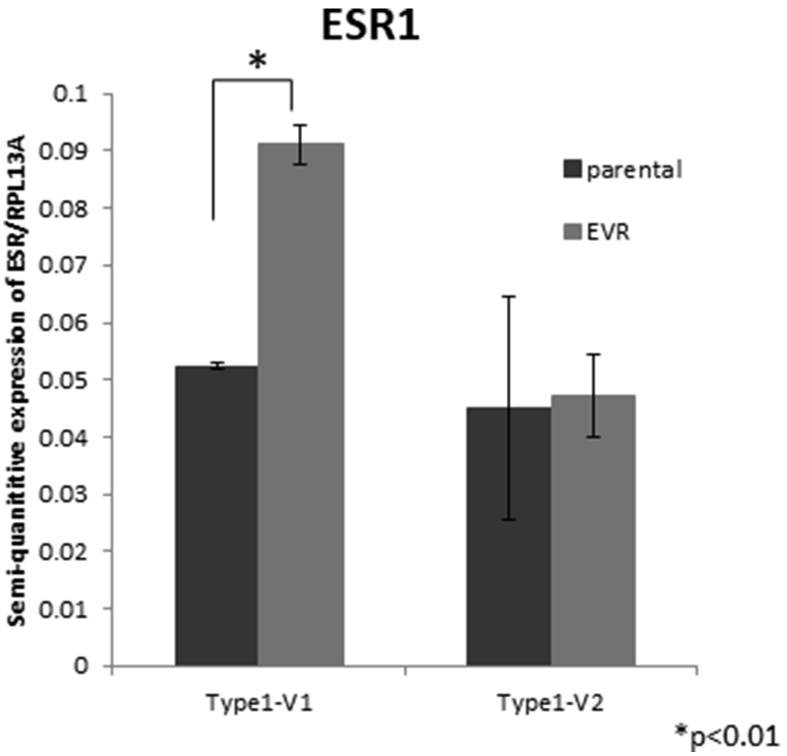

Supplementary Figure 4: *ESR1* expression in Type 1 EDR (V1, V2) and EvR cells. Data are shown as means ± SD of three independent experiments. *P<0.01 between parental and EvR of Type 1 EDR-V1 cells.

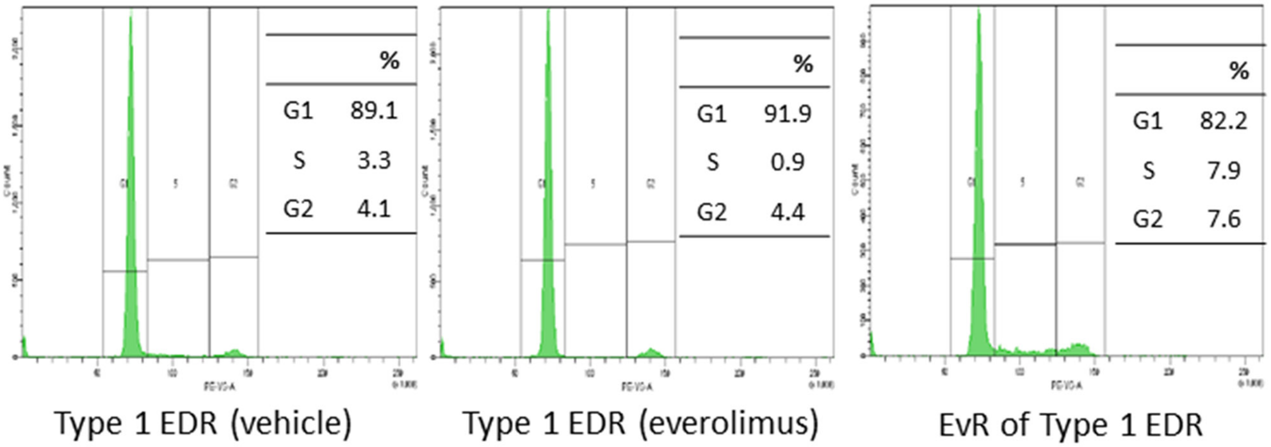

Supplementary Figure 5: Cell cycle fluorescence-activated cell sorter analysis. Type 1 EDR (V1) cells were treated with vehicle for 24 h. (left). Type 1 EDR (V1) cells were treated with everolimus (20 nM) for 24 h (middle). EvR-v1 cells under the usual harvested conditions (right). EvR cells were found to be in S phase more frequently than parental EDR cells.

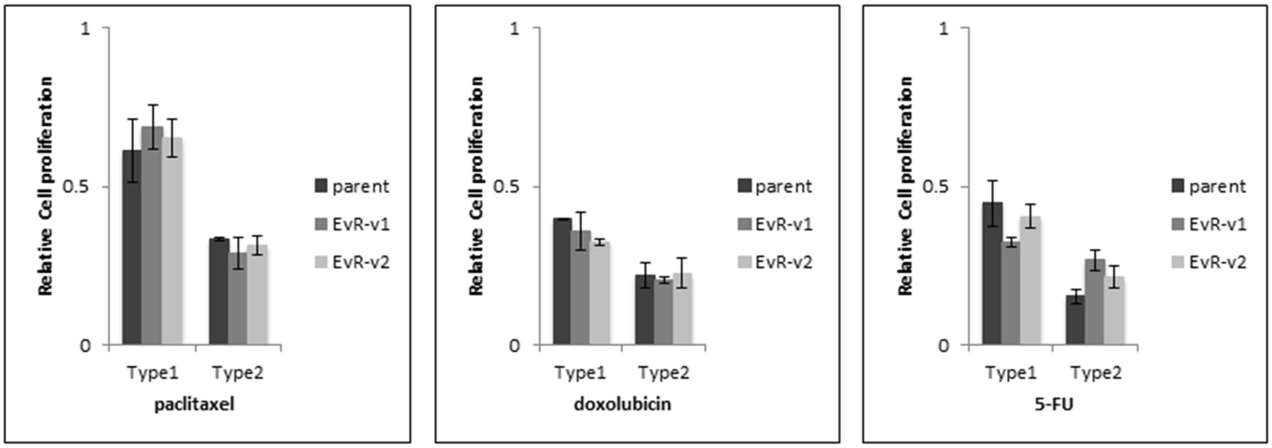

Supplementary Figure 6: Responses of various chemotherapeutic agents in Type 1-V1 or 2 EDR-V1 cells and in the EvR variants. Agent concentrations were as follows: paclitaxel, 1 nM; doxorubicin, 40 nM; 5-FU, 3 μM. Data are indicated as values relative to cells treated with vehicle. All data are shown as means ± SD of three independent experiments.
